# Outcomes of pediatric severe traumatic brain injury patients treated in adult trauma centers with and without added qualifications in pediatrics — United States, 2009

**DOI:** 10.1186/2197-1714-1-15

**Published:** 2014-06-02

**Authors:** Fernando Ovalle, Likang Xu, William S Pearson, Bridget Spelke, David E Sugerman

**Affiliations:** 1 Division of Injury Response, National Center for Injury Prevention and Control, Centers for Disease Control and Prevention, 4470 Buford Highway NE, MS-F62, Atlanta, GA 30341 USA; 2 Vanderbilt University School of Medicine, Nashville, TN USA

**Keywords:** Traumatic brain injury, Pediatrics, Trauma centers, Mortality

## Abstract

**Background:**

Pediatric traumatic brain injury (TBI) is an important public health problem and little is known about site of care and outcomes of children with severe TBI. Across the country, most injured children are treated in adult trauma centers (ATCs). Recent literature suggests that ATCs with added qualifications in pediatrics (ATC-AQs) can have improved outcomes for pediatric trauma patients overall. This study characterizes the population of pediatric severe TBI patients treated at ATCs and investigates the effect of treatment at ATC-AQs versus ATCs on mortality.

**Methods:**

Using the 2009 National Trauma Data Bank, pediatric (age 0–17 years old) severe TBI (head Abbreviated Injury Scale score ≥3) patient visits at level I and II ATCs and ATC-AQs were analyzed for patient and hospital characteristics. The primary outcome was in-patient mortality. Multivariate analysis was performed on propensity score weighted populations to investigate effect of treatment at ATC-AQs versus ATCs on survival.

**Results:**

A total of 7,057 pediatric severe TBI patient visits in 398 level I and II trauma centers were observed, with 3,496 (49.5%) at ATC-AQs and 3,561 (50.5%) at ATCs. The mortality rate was 8.6% at ATC-AQs versus 10.3% at ATCs (*p* =0.0144). After adjusting for differences in case mix, patient, and hospital characteristics, mortality was not significantly different for patients treated in ATC-AQs versus ATCs (aOR = 0.896, 95% CI = 0.629–1.277). Mortality was significantly associated with age, length of hospital stay, firearm injury, GCS score, and head AIS (*p* <0.0001). Higher mortality odds were also associated with being uninsured (aOR = 2.102, 95% CI = 1.159–3.813), and the presence of additional non-TBI severe injuries (aOR = 1.936 95% CI = 1.175-3.188).

**Conclusions:**

After defining comparable populations, this study demonstrated no significant difference in mortality for pediatric severe TBI patients treated at ATC-AQs versus ATCs. Being younger, uninsured, and having severe injuries was associated with increased mortality. This study is limited by the exclusion of transferred patients and potentially underestimates differences in outcomes. Further research is needed to clarify the role of ATC additional pediatric qualifications in the treatment of severe TBI.

**Electronic supplementary material:**

The online version of this article (doi:10.1186/2197-1714-1-15) contains supplementary material, which is available to authorized users.

## Background

In the United States, traumatic brain injury (TBI) is the single leading cause of injury-related death and acquired disability among children and young adults, affecting both sexes and all economic, racial, and social backgrounds (Coronado et al. 2011; Keenan and Bratton 2006; Langlois et al. 2005). Between 2002–2006, for pediatric patients aged 0–19 years old there were nearly 700,000 TBIs alone or in conjunction with other injuries or conditions resulting in TBI-related emergency department (ED) visits, hospitalizations, and deaths (Faul et al. 2010). Most TBIs are mild (Glasgow Coma Scale [GCS] score 13-15); however, up to a quarter are moderate (GCS 9–12) or severe (GCS 3–8) and responsible for a disproportionately high degree of morbidity and mortality (National Center for Injury Prevention and Control 2003). Severely injured children are known to face higher rates of in-hospital mortality, longer hospital stays, and require extensive and specialized rehabilitation to address major cognitive sequelae (Densmore et al. 2006; Rivara et al. 2011; Slomine et al. 2006; Tepas et al. 2009; Yeates et al. 2004). Pediatric TBI also contributes to the healthcare cost burden in the United States, accounting for over $1 billion in total annual hospital charges (Schneier et al. 2006). With high rates of TBI-related morbidity and mortality contributing to many lost years of productive life, both the financial and human cost of pediatric TBI make it a significant public health problem.

Trauma systems in the U.S. evolved to improve the triage and treatment of injured patients, ultimately resulting in decreased mortality at trauma centers compared to non-trauma centers (MacKenzie et al. 2006). Pediatric trauma patients can have needs that differ from those of adult trauma patients, and the lack of pediatric-specific personnel and equipment at adult trauma centers (ATCs) that care for injured children has been documented (Athey et al. 2001; Gausche-Hill et al. 2007). On the other hand, pediatric trauma centers (PTCs) are specifically equipped and staffed with specialty-trained caregivers to provide optimal care for injured children (American College of Surgeons Committee on Trauma 2006). While some authors dispute outcome differences between ATCs and PTCs (Fortune et al. 1992; Jubelirer et al. 1990; Kaufmann et al. 1989; Knudson et al. 1992; Osler et al. 2001; Rhodes et al. 1993), other studies have demonstrated improvement in morbidity and mortality when pediatric trauma patients are treated at PTCs (Densmore et al. 2006; Mooney et al. 2006; Potoka et al. 2001; Pracht et al. 2008).

Due to the shortage of PTCs as well as geographic distributions limiting access to care at PTCs in many areas of the country (Nance et al. 2009), the majority of pediatric trauma patients in the U.S. receive care at ATCs (Segui-Gomez et al. 2003). PTCs alone cannot fully address the care of all injured children; instead, they must work in partnership with ATCs to optimize the system of care of pediatric trauma patients (Athey J et al. 2001). One development to the trauma system classification scheme is the ability of adult trauma centers to gain added qualifications in pediatrics (ATC-AQs) and therefore offer more directed care for pediatric patients. While the literature on ATC-AQs is limited, some studies have demonstrated improved outcomes for pediatric trauma patients compared to ATCs alone (Oyetunji et al. 2011; Potoka et al. 2000) and comparable outcomes with PTCs (Potoka et al. 2000).

Although it has been proposed that ATC-AQs improve overall outcomes for pediatric trauma patients compared to ATCs, little is known about the optimal triage strategy and the resulting outcomes of children with TBI, especially those with severe TBI who need high acuity care the most. Current guidelines and recommendation standards cite the limited evidence available in this area, especially on a national level (Adelson et al. 2003; Kochanek et al. 2012); therefore, further understanding of the effect of trauma systems on the care of children with severe TBI is needed to reduce the impact of this important public health issue. In order to address this gap, the objectives of our study were to characterize the population of pediatric severe TBI patients treated at ATC-AQs and ATCs, to investigate the effect of treatment at ATC-AQs versus ATCs on patient mortality and to inform pediatric patient transport decision-making, following severe TBI, in the absence of a PTC.

## Methods

The study design was a retrospective secondary analysis using the 2009 National Trauma Data Bank (NTDB) Research Data Set (RDS). This dataset is managed by the American College of Surgeons (ACS) and, in 2009, collected trauma visits voluntarily submitted by 682 U.S. trauma centers. The study population was comprised of visits of pediatric patients who were aged 0–17 years old with a diagnosis of severe TBI (defined by head maximum Abbreviated Injury Scale [AIS] score ≥3). TBI was identified using International Classification of Diseases, 9^th^ Revision Clinical Modification (ICD–9-CM) codes obtained from the NTDB. The Centers for Disease Control and Prevention (CDC) definition of TBI was used (Faul et al. 2010) and included the following codes: fracture of the vault or base of skull (800.0–801.9), other and unqualified multiple fractures of the skull (803.0–804.9), intracranial injury, including concussion, contusion, laceration, and hemorrhage (850.0–854.1), injury to optic nerve and pathways (950.1–950.3), shaken infant syndrome (995.55), and head injury, unspecified (959.01).

**Figure 1:**
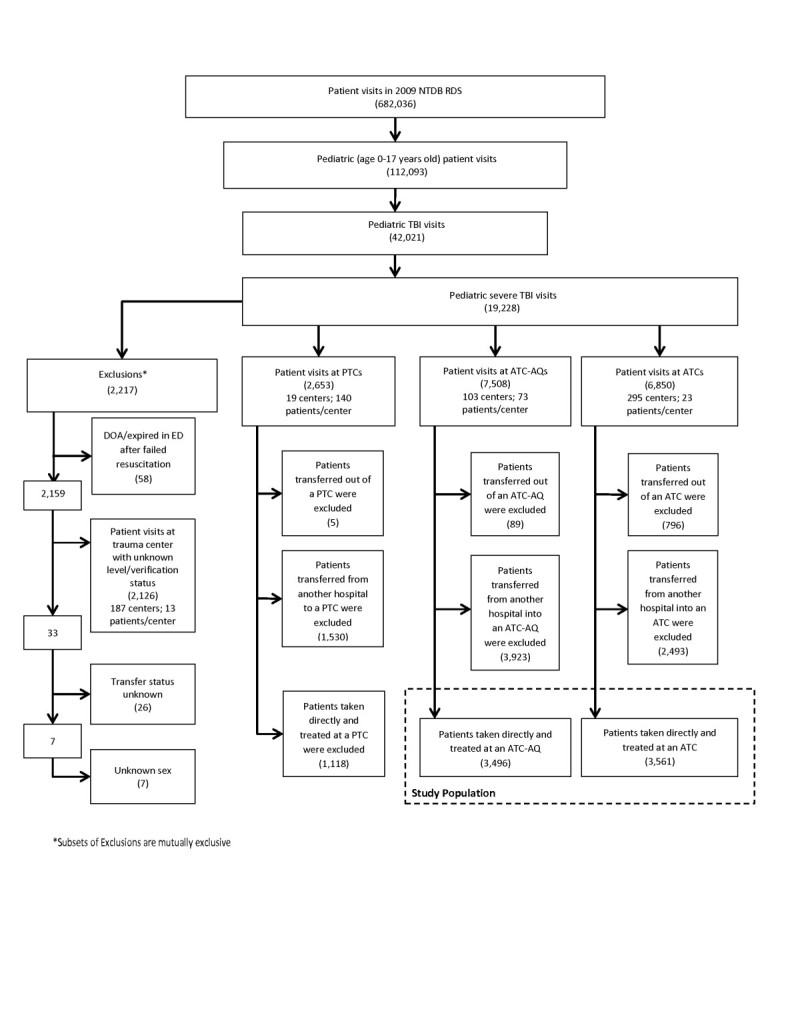
**Derivation of pediatric severe TBI study population using 2009 National Trauma Data Bank (NTDB) Research Data Set (RDS).**

Patients who were dead on arrival or expired in the ED after failed resuscitation, patient visits at trauma centers with unknown level, and those with unknown sex were excluded (Figure 1). The location of care at a PTC, an ATC-AQ or an ATC without pediatric sub-specialization was investigated. Using NTDB facility variables (American College of Surgeons Committee on Trauma 2008), a PTC was defined as having an ACS-verified adult trauma center or state-designated adult trauma center level of “not applicable” with additional level I or II ACS pediatric verification or level I or II state pediatric designation. An ATC was defined as a level I or II ACS-verified adult trauma center or a level I or II state-designated adult trauma center. An ATC-AQ was defined as a level I or II ACS-verified adult trauma center with additional ACS pediatric verification or a level I or II state-designated adult trauma center with additional state pediatric designation. In two cases where ACS verification and state designation were both present and differed on level of trauma center, the ACS specification was used. All patients transferred into ATC-AQs and ATCs were excluded from analysis in order to minimize bias arising from severely injured patients who were transferred to a higher level or more specialized centers. Transfers out were excluded as their outcomes cannot be determined in the NTDB (Figure 1). Repeated analysis on transfers alone and including these subpopulations revealed similar results (data not shown). Lastly, because pediatric verification/designations are only available for level I or II centers in the NTDB, all visits seen at non-level I or II adult trauma centers were excluded.

Definitions of the variables examined are described in the NTDB Data Dictionary (American College of Surgeons Committee on Trauma 2008). The primary outcome was in-hospital mortality. Unadjusted analysis included the secondary outcome measures length of stay (LOS) in days, ICU days, ventilator days, hospital complications (any medical complication during the hospital stay as defined by the NTDB), and hospital discharge disposition among survivors (classified as home with no home services/extra care versus rehabilitation/other care facilities [including short-, long-, and intermediate-care facilities, home with home health services, and hospice] as well as other/unknown [including left against medical advice]). Other variables included in both unadjusted and adjusted analyses were age (infants/toddlers aged 0–3 years old, preschool/young children aged 4–8 years old, preadolescents aged 9–13 years old, and adolescents aged 14–17 years old), sex, race/ethnicity (white, African American, Hispanic, other [including Asian, Native American, and Hawaiian/Pacific Islander] and unknown), trauma center level (I or II), Injury Severity Score (ISS) as determined in the NTDB by ICDMAP-90 calculation (severe [ISS >15] or non-severe [ISS ≤15]), emergency department Glasgow Coma Scale (ED GCS 3-4, 5-6, 7-8, >8), insurance status (commercial and other, self-pay, Medicaid, and unknown), mechanism of injury (falls, firearms, motor vehicle accident [MVA], struck by/against, all other mechanisms of injury described by the NTDB, and unknown), head AIS (3, 4, 5, or 6), TBI type (subdural hematoma (SDH), subarachnoid hemorrhage (SAH) or extradural hematoma (EDH); unspecified intracranial hemorrhage (ICH); cerebral laceration or contusion; other intracranial injury; closed head injury without mention of intracranial injury; or “other” for cases not meeting criteria defined above), and whether the patient had isolated versus polytraumatic severe TBI. In order to better study the effect of other severe injuries on outcomes, the polytraumatic severe TBI population was further subdivided into non-severe polytraumatic (severe TBI plus any injury in a body region other than the head with AIS score of <3) and severe polytraumatic (severe TBI plus any injury in a body region other than the head with AIS score of ≥3) groups.

All data analysis was performed using SAS 9.3 (SAS Institute Inc., Cary, NC). Unadjusted bivariate analyses were performed on patient and injury characteristic variables in both ATC and ATC-AQ groups. Two-tailed independent *t* tests were used to compare continuous variables and *χ*^2^ tests were used to compare categorical variables. Multiple imputation techniques were used to preserve sample size and account for missing values in our sample. Data were missing for fewer than 5% of patient visits with the exception of insurance (12.6% missing) and ISS (8.9% missing data). Ten imputed data sets were created. For each data set, generalized estimating equations (GEEs) were used to adjust for clustering within sites (MacKenzie et al. 2006; Roudsari et al. 2008). Data sets were analyzed in parallel and the results were combined using standard techniques (Roudsari et al. 2008).

To account for potential confounding factors and study the effect of treatment center type on outcome, the inverse probability of treatment weighting, or propensity score weighting, was used to create balanced and comparable populations in our two study groups (patients treated at ATCs and ATC-AQs) (Cudnik et al. 2009; MacKenzie et al. 2006). Including the primary outcome, the following variables were included in the development of the propensity score: age, sex, race, insurance status, trauma center level, ED GCS, mechanism of injury, presence of TBI polytrauma, head AIS, TBI type, and length of stay (LOS).

Multivariate logistic regression was used to examine the effect of independent variables on patient survival to discharge. Adjusted odds ratio (aOR) and 95% confidence interval (CI) for in-hospital mortality were calculated after adjusting for sex, age, race/ethnicity, insurance, trauma center level, ED GCS, head AIS, mechanism of injury, presence of TBI polytrauma and TBI type. Age, LOS and GCS variables were used as continuous variables in the multivariate analysis instead of their categorical breakdowns. Significance level for all statistical tests was set at *p* <0.05.

## Results

Out of 682,036 trauma center patient visits reported in the 2009 NTDB RDS, there were 112,093 (16.4%) patient visits by pediatric patients who were aged 0–17 years old (Figure 1). Out of these, a total of 42,021 visits (37.5% of total pediatric patient visits) were found that included a TBI diagnosis, and 19,228 (45.8% of total pediatric TBI visits) were found to have severe TBI. There were 2,217 patient visits (11.5% of pediatric severe TBI visits) excluded from our study due to death on arrival or prior to ED discharge, unknown level of trauma center, unknown transfer status, or unknown patient sex. Patient visits at PTCs (2,653), including transfers, were also excluded. Our final study population drew from 14,358 pediatric severe TBI patient visits seen at 398 level I and II trauma centers. All patients transferred into or out of an ATC-AQ or ATC were excluded. Our final study population included 7,057 severe pediatric TBI visits (Figure 1).

Of 7,057 pediatric severe TBI patient visits, 3,496 (49.5%) were treated at ATC-AQs and 3,561 (50.5%) were at ATCs (Table 1). Compared to ATCs, ATC-AQs were found to have an overall younger population (*p* <0.0001), more African American patient visits (*p* = 0.0002), more Medicaid patient visits (*p* < 0.0001), and more patient visits at level I centers (*p* <0.0001). With respect to mechanism of injury, ATC-AQs were found to have significantly fewer firearm-related (*p* <0.0001), motor vehicle (*p* <0.0001) and struck by/against visits (*p* < 0.0001) than ATCs. Compared to ATCs, ATC-AQs were found to have fewer visits with severe polytraumatic TBI (*p* = 0.0002) and more visits with other intracranial injury (*p =* 0.0001). No statistically significantly differences between the two categories of centers were noted by sex or ISS (Table 1).

**Table 1:** **Patient and injury characteristics of pediatric severe TBI patient visits treated in ATC-AQs and ATCs**

	ATC-AQ	ATC	Total	Odds Ratio ^*^(95% CI)	P value ^*^
	n (%)	n (%)	n (%)		
	(N = 3,496)	(N = 3,561)	(N = 7,057)		
**Sex**					
Female	1,215 (34.8)	1,144 (32.1)	2,359 (33.4)	1.0 (reference)	
Male	2,281 (65.2)	2,417 (67.9)	4,698 (66.6)	0.889 (0.805–0.981)	0.0202
**Age group (years)**					
0–3	1,154 (33.0)	681 (19.1)	1,835 (26.0)	1.0 (reference)	
4–8	604 (17.3)	426 (12.0)	1,030 (14.6)	0.837 (0.716–0.978)	0.0279
9–13	618 (17.7)	539 (15.1)	1,157 (16.4)	0.677 (0.583–0.786)	<.0001
14–17	1,120 (32.0)	1,915 (53.8)	3,035 (43.0)	0.345 (0.306–0.389)	<.0001
**Race/Ethnicity**					
White	1,895 (54.2)	2,027 (56.9)	3,922 (55.6)	1.0 (reference)	
Black	606 (17.3)	500 (14.0)	1,106 (15.7)	1.296 (1.134–1.482)	0.0002
Hispanic	622 (17.8)	648 (18.2)	1,270 (18.0)	1.027 (0.905–1.165)	0.6983
Other	259 (7.4)	251 (7.0)	510 (7.2)	1.104 (0.918–1.327)	0.3005
Unknown	114 (3.3)	135 (3.8)	249 (3.5)	0.903 (0.699–1.168)	0.4720
**Insurance**					
Commercial and other	1,709 (48.9)	1,941 (54.5)	3,650 (51.7)	1.0 (reference)	
Self Pay	233 (6.7)	314 (8.8)	547 (7.8)	0.843 (0.703–1.011)	0.0660
Medicaid	1,047 (29.9)	925 (26.0)	1,972 (27.9)	1.286 (1.152–1.435)	<.0001
Unknown	507 (14.5)	381 (10.7)	888 (12.6)	1.511 (1.304–1.752)	<.0001
**Trauma center level**					
Level I	2,482 (71.0)	2,228 (62.6)	4,710 (66.7)	1.0 (reference)	
Level II	1,014 (29.0)	1,333 (37.4)	2,347 (33.3)	0.683 (0.618–0.754)	<.0001
**ED GCS**					
3–4	670 (19.2)	714 (20.1)	1,384 (19.6)	0.913 (0.81–1.028)	0.1361
5–6	94 (2.7)	135 (3.8)	229 (3.2)	0.677 (0.518–0.887)	0.0045
7–8	111 (3.2)	156 (4.4)	267 (3.8)	0.692 (0.539–0.889)	0.0038
>8	2,499 (71.5)	2,431 (68.3)	4,930 (69.9)	1.0 (reference)	
Unknown	122 (3.5)	125 (3.5)	247 (3.5)	0.949 (0.735–1.226)	0.6962
**Head AIS**					
3	1,125 (32.2)	1,162 (32.6)	2,287 (32.4)	1.0 (reference)	
4	2,021 (57.8)	1,873 (52.6)	3,894 (55.2)	1.115 (1.005–1.236)	0.0399
5	345 (9.9)	515 (14.5)	860 (12.2)	0.692 (0.590–0.811)	<.0001
6	5 (0.1)	11 (0.3)	16 (0.2)	0.469 (0.163–1.356)	0.2098
**ISS Score**					
ISS < 15	847 (24.2)	875 (24.6)	1,722 (24.4)	1.0 (reference)	
ISS > 15	2,329 (66.6)	2,378 (66.8)	4,707 (66.7)	1.012 (0.906–1.130)	0.8437
Unknown	320 (9.2)	308 (8.6)	628 (8.9)	1.073 (0.894–1.289)	0.4560
**Mechanism of injury**					
Falls	1,028 (29.4)	858 (24.1)	1,886 (26.7)	1.0 (reference)	
Firearms	108 (3.1)	185 (5.2)	293 (4.2)	0.487 (0.378–0.628)	<.0001
Motor vehicles	1,303 (37.3)	1,433 (40.2)	2,736 (38.8)	0.759 (0.675–0.854)	<.0001
Struck by/against	328 (9.4)	407 (11.4)	735 (10.4)	0.673 (0.567–0.798)	<.0001
Other	728 (20.8)	678 (19.0)	1,406 (19.9)	0.896 (0.780–1.029)	0.1289
Unknown	1 (0.0)	0 (0.0)	1 (0.0)		
**TBI type**					
SAH, SDH, EDH	811 (23.2)	788 (22.1)	1,599 (22.7)	1.171 (1.036–1.325)	0.0121
Intracranial Hemm (other and unspecified)	107 (3.1)	122 (3.4)	229 (3.2)	0.998 (0.762–1.308)	1.0000
Cerebral Laceration and Contusion	246 (7.0)	260 (7.3)	506 (7.2)	1.077 (0.891–1.302)	0.4680
Other Intracranial Injury	169 (4.8)	118 (3.3)	287 (4.1)	1.630 (1.274–2.086)	0.0001
No Mention of Intracranial Injury	853 (24.4)	782 (22.0)	1,635 (23.2)	1.242 (1.099–1.403)	0.0005
Other	1,310 (37.5)	1,491 (41.9)	2,801 (39.7)	1.0 (reference)	
**Presence of severe TBI polytrauma** ^**†**^					
TBI only	1,265 (36.2)	1,140 (32.0)	2,405 (34.1)	1.0 (reference)	
TBI + non–Severe Injury	1,500 (42.9)	1,580 (44.4)	3,080 (43.6)	0.856 (0.769–0.952)	0.0043
TBI + Severe Injury	731 (20.9)	841 (23.6)	1,572 (22.3)	0.783 (0.690–0.890)	0.0002
**Hospital complications** ^**‡**^					
No	1,716 (49.1)	1,448 (40.7)	3,164 (44.8)	1.0 (reference)	
Yes	333 (9.5)	423 (11.9)	756 (10.7)	0.664 (0.566–0.779)	<.0001
Unknown	1,447 (41.4)	1,690 (47.5)	3,137 (44.5)	0.722 (0.654–0.798)	<.0001
**Discharge disposition** ^**§**^					
Home (without extra care)	2,621 (82.0)	2,382 (74.6)	5,003 (78.3)	1.0 (reference)	
Rehabilitation/other care facility^¶^	512 (16.0)	674 (21.1)	1,186 (18.6)	0.690 (0.608–0.784)	<.0001
Other/unknown	64 (2.0)	139 (4.4)	203 (3.2)	0.418 (0.310–0.566)	<.0001
**ICU Days (Mean ± SE)**	4.84 (0.16)	5.01 (0.17)	4.93 (0.12)		0.4684**
**Length of stay (days) (Mean ± SE)**	6.17 (0.16)	6.09 (0.17)	6.13 (0.12)		0.7369**
Alive (Mean ± SE)	6.40 (0.17)	6.44 (0.19)	6.42 (0.13)		0.8665**
Died (Mean ± SE)	3.73 (0.4)	3.04 (0.27)	3.35 (0.23)		0.1574**
**Ventilator days (Mean ± SE)**	6.09 (0.29)	5.57 (0.26)	5.81 (0.19)		0.1728**
**Mortality**	299 (8.6)	366 (10.3)	665 (9.4)	0.816 (0.695–0.959)	0.0144

Data are reported as counts, percentages (%), and mean + SE.

^*^Calculated by *χ*^2^ test; **Calculated by two-tailed independent *t*-test.

^**†**^Non-severe polytraumatic TBI = severe TBI plus injury in body region other than head with AIS score < 3, severe polytraumatic TBI = severe TBI plus injury in body region other than head with AIS score ≥3.

^**‡**^Defined by NTDB as any medical complication occurring during hospital stay.

^**§**^Disposition does not include deaths (n = 665).

^**¶**^Other care facilities include short-, long-, and intermediate-care facilities, home with home health services/extra care, and hospice.

The study population included 665 deaths. More specifically, ATC-AQs were found to have 299 deaths compared to 366 deaths at ATCs (8.6% vs. 10.3% mortality rate, respectively; *p* = 0.0144; Table 1). Patients treated at ATC-AQs were less likely to have in-hospital complications (*p* <0.0001). No significant difference in the mean length of stay, in the mean number of ICU days, and the mean number of ventilator days was seen between the centers. Propensity score adjustment created equal populations for all patient and injury characteristics except insurance status, which remained significantly different for both groups (data not shown).

After adjusting for differences in case mix using propensity scoring, multiple logistic regression analysis adjusted for treatment center type and all variables in Table 2 found no significant difference in mortality between ATC-AQs and ATCs (aOR = 0.896; 95% CI 0.629–1.277) (Table 2). Mortality odds were significantly associated with being younger, having a shorter length of stay, being uninsured, having a firearm injury, and the presence of an additional severe traumatic injury (Table 2). Having a fall and a struck by/against injury were associated with lower mortality odds. Patient visits that were self-pay had significantly higher mortality than insured visits (aOR = 2.102; 95% CI 1.159–3.813). Mortality was also higher with increasing head AIS and decreasing GCS score (Table 2).

**Table 2:** **Multivariate model of pediatric severe TBI patient mortality** ^*^

	aOR (95% CI)	P value
**Age**	0.936 (0.907–0.966)	<.0001
**Length of hospital stay (days)**	0.821 (0.762–0.883)	<.0001
**GCS score**	0.633 (0.600–0.668)	<.0001
**Trauma center**		
ATC–AQ	1.0 (reference)	
ATC	0.896 (0.629–1.277)	0.5446
**Sex**		
Female	1.0 (reference)	
Male	1.249 (0.898–1.738)	0.1857
**Race/Ethnicity**		
White	1.0 (reference)	
Black	1.562 (0.967–2.524)	0.0683
Hispanic	1.284 (0.876–1.881)	0.1998
Other	1.914 (0.986–3.714)	0.0550
**Insurance**		
Commercial and other	1.0 (reference)	
Self Pay	2.102 (1.159–3.813)	0.0145
Medicaid	1.241 (0.824–1.870)	0.3006
**Trauma center level**		
Level I	1.0 (reference)	
Level II	1.155 (0.797–1.675)	0.4471
**Head AIS**		
3	1.0 (reference)	
4	4.113 (2.406–7.033)	<.0001
5	32.231 (17.580–59.094)	<.0001
6	22.641 (2.521–203.370)	0.0053
**Mechanism of injury**		
Falls	0.333 (0.173–0.644)	0.0011
Firearms	7.780 (3.493–17.328)	<.0001
Motor vehicles	1.251 (0.807–1.940)	0.3175
Struck by/against	0.399 (0.194–0.821)	0.0125
Other	1.0 (reference)	
**TBI type**		
SAH, SDH, EDH	0.797 (0.525–1.212)	0.2891
Intracranial Hemm (other and unspecified)	1.024 (0.446–2.352)	0.9555
Cerebral Laceration and Contusion	0.929 (0.541–1.595)	0.7881
Other Intracranial Injury	1.675 (0.906–3.098)	0.1001
No Mention of Intracranial Injury	1.175 (0.689–2.001)	0.5538
Other	1.0 (reference)	
**Presence of severe TBI polytrauma**		
TBI only	1.0 (reference)	
TBI + non–severe injury	0.681 (0.428–1.083)	0.1043
TBI + Severe injury	1.936 (1.175–3.188)	0.0095

*Analysis sample derived from multiple imputation techniques. Treatment groups at ATC-AQs and ATCs adjusted with propensity score weighting.

## Discussion

This study characterizes children who sustained a severe TBI and were treated at level I and II adult trauma centers either with or without added qualifications in pediatrics and provides a large adjusted multivariate analysis of in-hospital mortality by type of adult trauma center. Overall, ATC-AQs had a younger patient population, fewer uninsured visits, and more fall-related visits than ATCs. Consistent with prior studies, mortality odds after severe TBI are higher for uninsured patients (Haider et al. 2008; Rosen et al. 2009) and firearm injuries (Asemota et al. 2013; Deans et al. 2013; Sugerman et al. 2012). As expected, mortality is associated with injury severity indexes including head AIS and ED GCS (Keenan and Bratton 2006). Younger age and the presence of a severe polytraumatic injury were also associated with increased mortality odds. However, children treated at ATC-AQs were no more likely to die than children treated at ATCs. In this light, our results substantiate previous findings of equivalency in survival outcomes between pediatric-specialized and non-pediatric-specialized adult trauma centers for certain populations of injured pediatric patients (Fortune et al. 1992; Jubelirer et al. 1990; Kaufmann et al. 1989; Knudson et al. 1992; Osler et al. 2001; Rhodes et al. 1993) and indicates that there is no increased risk of in-patient mortality for severe pediatric TBI patients triaged to an adult trauma center.

The highest level of care for injured children in U.S. is the stand-alone PTC, which has the full array of pediatric specialists and services, as well as an institutional-wide commitment to pediatric injury education, research, and prevention. While multiple studies have demonstrated PTCs’ favorable effects on mortality rates (Densmore et al. 2006; Mooney et al. 2006; Potoka et al. 2001; Pracht et al. 2008), most pediatric trauma patients are treated in ATCs (Segui-Gomez et al. 2003). According to the American College of Surgeons Committee on Trauma, to gain the “added qualifications in pediatrics” label, an ATC must admit ≥100 children <15 years of age; have a pediatric surgeon credentialed for pediatric trauma care; and have a pediatric emergency department/intensive care area with appropriate resuscitation equipment (American College of Surgeons Committee on Trauma 2006). As opposed to general trauma managed by pediatric general surgeons, children with severe neurotrauma often require pediatric neurosurgery and neurointensivists care (Kochanek et al. 2012) only found in PTCs (American College of Surgeons Committee on Trauma 2006). The lack of specialized pediatric neurological care at both ATCs and ATC-AQs may explain why no difference in mortality was seen among pediatric severe TBI patients treated at these centers.

The Brain Trauma Foundation (BTF) has outlined the acute management of pediatric severe TBI patients (Adelson et al. 2003; Kochanek et al. 2012) and makes three levels of recommendations – standards, guidelines, and options. As noted by the BTF, there is currently insufficient evidence to establish a standard with regards to the triage of pediatric severe TBI patients. At the guideline level, the BTF recommends that in a metropolitan area, children with severe TBI should be transported directly to a PTC if available. At the option level, they suggest triage to an ATC-AQ in preference to a level I or II ATC without added qualifications. These recommendations were evaluated as “weak” by the BTF, with the option to preferentially triage pediatric severe TBI patients to ATC-AQs over ATCs primarily based on the availability of a single retrospective study (Potoka et al. 2000). This study, which did not account for differences in baselines characteristics between groups or utilize multivariate statistics, demonstrated higher survival for severe TBI patients treated in PTCs or ATC-AQs than those in ATCs across Pennsylvania. Another study which was not included in the BTF evidence list utilized the 2002–2006 NTDB and suggested a lower mortality for pediatric severe TBI patients treated at Level I ATC-AQs compared to Level I ATCs, though this result only approached significance (*p* = 0.05) (Oyetunji et al. 2011). Three factors likely affected these mortality differences compared to our results: the omission of children with severe TBI treated at level II ATCs (nearly 40% of our total cases), our use of the CDC definition of severe TBI (Oyetunji et al. relied on GCS > 8) and the use of propensity score weighting to create comparable populations.

While the literature regarding the triage of pediatric severe TBI patients to ATC-AQs versus ATCs is limited, if there are no differences in mortality between two types of centers, the ultimate triage destination decision should be made in conjunction with current guidelines. The CDC Guidelines for Field Triage of Injured Patients (Sasser et al. 2012) recommends transport of all patients with moderate or severe TBI (GCS ≤ 13) to the highest level of care within the trauma system, regardless of AQ designation. Further studies on outcomes by triage decision are warranted to inform the design of pediatric trauma care delivery systems. Facilities treating children injured with severe TBI should have available prompt CT scanning, neurosurgical care, and the ability to monitor intracranial pressure and manage intracranial hypertension. Certainly, centers with added pediatric specialization have added benefits for many subpopulations of injured children, specifically those with abdominal blunt-force injuries, for whom triaging to the highest level of pediatric specialized care available is more widely cited (Densmore et al. 2006; Mooney et al. 2006; Oyetunji et al. 2011; Potoka et al. 2001; Potoka et al. 2000; Pracht et al. 2008).

This study has several limitations. The NTDB includes only in-hospital outcomes, not capturing long-term disability for those discharged or transferred out and therefore limiting our analysis to in-patient mortality and potentially underestimating differences in outcomes. Stand-alone PTCs were not well represented in this voluntary database. Although it was assumed that with specialized pediatric neurosurgical and intensivist coverage that patient outcomes would be improved in PTCs, targeted research is lacking, and it remains the case that most pediatric trauma patients are treated in ATCs. Our study does not address the quality or standard of care at ATCs: namely, if the differing facility types are equally effective or equally ineffective in their treatment of this pediatric population. Further studies are needed to determine whether treatment at ATC-AQs provides a survival advantage to patients with severe TBI by reducing long-term disability. Finally, the NTDB-RDS is a retrospective and voluntary visit-based database and the variation in completeness between centers could lead to potential bias. Furthermore, the RDS is a non-representative sample limiting the generalizability of these results. Despite these well-established limitations of the NTDB, to our knowledge, this is the first study that determines survival outcomes by ATC type with advanced statistical techniques to address the concerns of missing data, clustering by facility and the non-random distribution of cases between center types.

## Conclusion

Pediatric severe TBI patient visits at ATC-AQs have similar in-hospital survival odds relative to ATCs. Consistent with prior studies, mortality odds after severe TBI are higher for uninsured patients, younger children, firearm injuries, and the presence of an additional severe traumatic injury. When lack of access to care at a PTC necessitates a triage decision between different types of adult trauma centers, these findings support prioritizing patient triage to the highest level center available, consistent with the CDC Guidelines for Field Triage of Injured Patients (Sasser et al. 2012). These findings are limited by the exclusion of severe TBI patients transferred to another facility for treatment. Though this enables the development of comparable patient groups, it increases the potential for underestimating differences in outcomes. Ultimately, ongoing research and surveillance efforts are needed to improve pediatric trauma triage in the U.S.

## Authors’ original submitted files for images

Below are the links to the authors’ original submitted files for images. Authors’ original file for figure 1

## Abbreviations

TBI: Traumatic brain injury
ATCs: Adult trauma centers
ATC-AQs: Adult trauma centers with added qualifications in pediatrics
PTCs: Pediatric trauma centers
NTDB: National Trauma Data Bank
AIS: Abbreviated Injury Scale
ISS: Injury Severity Score
CDC: Centers for Disease Control and Prevention.
