# Supplementary figures and images for: Outcomes of pediatric severe traumatic brain injury patients treated in adult trauma centers with and without added qualifications in pediatrics — United States, 2009
Source: Inj Epidemiol. 2014 Jun 2;1(1):15. doi: 10.1186/2197-1714-1-15 (PMC5005579; doi:10.1186/2197-1714-1-15)

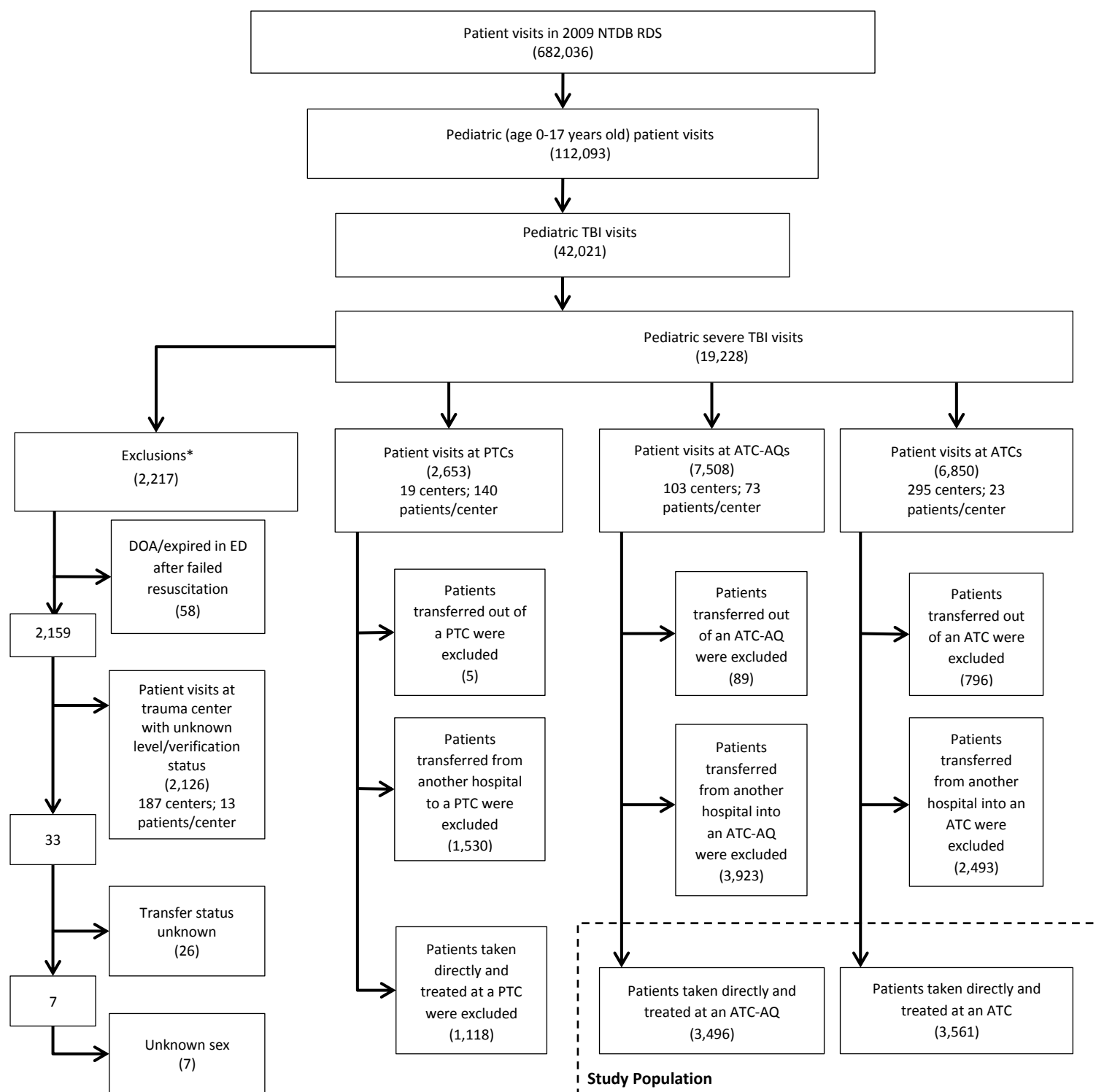

\*Subsets of Exclusions are mutually exclusive

Supplement: Supplementary file 1 — Authors’ original file for figure 1 [file 40621_2014_15_MOESM1_ESM.pdf]
